# A pathway-based association analysis model using common and rare variants

**DOI:** 10.1186/1753-6561-5-S9-S85

**Published:** 2011-11-29

**Authors:** Lu Cheng, Pingzhao Hu, Jenna Sykes, Melania Pintilie, Geoffrey Liu, Wei Xu

**Affiliations:** 1 Department of Biostatistics, Princess Margaret Hospital, 610 University Ave., Toronto, ON M5G 2M9, Canada; 2 Centre for Applied Genomics, Hospital for Sick Children Research Institute, 101 College Street, Toronto, ON M5G 1L7, Canada; 3 Dalla Lana School of Public Health, University of Toronto, 155 College St., Toronto, ON M5T 3M7, Canada

## Abstract

How various genetic effects in combination affect susceptibility to certain disease states continues to be a major area of methodological research. Various rare variant models have been proposed, in response to a common failure to either identify or validate biologically driven causal genetic variants in genome-wide association studies. Adopting the idea that multiple rare variants may effectively produce a combined effect equal to a single common variant effect through common linkage with this variant, we construct a pathway-based genetic association analysis model using both common and rare variants. This genetic model is applied to the disease status of unrelated individuals in replication 1 from Genetic Analysis Workshop 17. In this simulated example, we were able to identify several pathways that were potentially associated with the disease status and found that common variants showed stronger genetic effect than rare variants.

## Background

In the search for causal variants, an abundance of research has been focused on relatively frequent variants that are assumed to be located near the true causal variants. This focus is based on a popular hypothesis that common single-nucleotide polymorphisms (SNPs) contribute to the genetic effects underlying complex traits, where, traditionally, common SNPs are defined to be the ones with minor allele frequency greater than 1%. Genome-wide association study is an approach that scans markers across the whole genome. It has been proven to be quite successful, as more than 2,000 common variants have been identified to be associated with common diseases or related traits. Once the genetic markers have been found, extensive resequencing of the nearby sites is done to seek out the true causal SNPs. This step, however, has not been as successful as genome-wide association study, which raises the question of the common trait/common variant assumption [1-3]. Dickson et al. [2] proposed the synthetic rare variants effects model, which provides an alternative explanation to the significance of found markers. That is, the common variant association signal may actually represent effects from multiple rare variants, a situation that happens when the multiple rare variants occur, by chance, more frequently in association with one allele at the signal common SNP than with the other. Two scenarios are possible for the rare variant effect. In the first scenario, the true genetic effects are caused solely by rare SNPs; in the second scenario, both common and rare SNPs contribute to the disease status or related traits. To address this situation, we model the effects of rare and common variants both separately and in combination, with the common goal of uncovering the true genetic effects.

Because of the low penetrance of rare variants, the power to detect the effect of a single rare SNP is small. Modeling groups of rare SNPs is an efficient way to detect rare variant effects. Groups may refer to genes or pathways or other biologically meaningful units. A genetic pathway is a set of interactions between several genes that function together to affect a disease or trait [4]. Experiments from genome-wide genetic studies have demonstrated that modeling at the pathway level (i.e., considering genes within a pathway together) may improve the detection of genetic effects [4,5].

Using the mini-exome data provided by Genetic Analysis Workshop 17 (GAW17), we aim to analyze the effects from rare and common variants at the pathway level to identify potential disease-associated pathways.

## Methods

### Data description

The mini-exome data provided by GAW17 consists of 697 unrelated individuals from the 1000 Genomes Project. Phenotypes of these individuals include one binary trait of affection status and three continuous quantitative traits. Genetic information is available on 24,487 SNPs distributed across all autosomal chromosomes and comes from 3,205 genes. Clinical factors include age, sex, smoking status, and ethnic origin, where ethnic origin is reclassified into Asian, African, and European. Two hundred replications of the data were generated, with the genetic information, age, sex, and ethnic origin held fixed while the phenotype variables and smoking status were simulated across the 200 replications.

### Analysis

We used PLINK [6] to provide summary statistics for each of the SNPs, including minor allele frequency, genotype distribution, and Hardy-Weinberg equilibrium (HWE) test. The SNPs that failed the HWE test (*p* < 1 × 10^−6^) in each of the subpopulation groups were excluded from further analysis. We applied nonadjusted logistic regression models to each of the SNPs and the disease status before further modeling was carried out. To determine whether our models should adjust for the quantitative effects in addition to the other clinical factors, we explored the associations between the traits and the disease status using logistic regression.

For each individual *i*, *i* = 1, …, 697, we define the disease status as the outcome as follows:(1)

We applied the genetic model to the pathway level and defined risk scores representing either rare or common variant effects of each pathway using a collapsing methods (described in more detail later), where a threshold of 1% for the minor allele frequency was used to divide rare and common variants. We then combined the two different risk scores into a single risk score to assess whether a pathway was associated with the disease. We ran unadjusted and adjusted models. The unadjusted model uses only the genetic risk score in a model, and the adjusted model uses the covariates Age, Sex, Smoke, and the population in the model as well. To be more specific, we describe our modeling steps in what follows.

**Step 1.** Apply the unadjusted logistic regression model to the rare variants effect. We construct a risk score, defined as the count or proportion of minor alleles of the rare variants within a pathway, to represent the rare variants effect [7]. We denote this risk score RS_rare_.

**Step 2.** Apply the adjusted logistic regression model to the common variants effect. First, we use the least absolute shrinkage and selection operator (LASSO) [8] for logistic regression to select candidate common variants within each pathway, where the selection is done by always incorporating other covariates, including Age, Sex, Smoking status, population origin, and the three quantitative traits. That is, we fit:(2)

where *X_C_*_,_*_j_* is the *j*th common SNP in a specific pathway, and:(3)

represents the adjusted covariates in the model. The model selection is based on maximizing the corresponding likelihood function subject to ∑ *_j_* |*γ_j_*| <*λ*, where *λ* is a LASSO penalty parameter. Some of the estimated regression coefficients  can be 0, which implies that the corresponding common SNP does not contribute to the outcome. The process leads to variable selection of the associated common variants.

If none of the common variants are selected, then we conclude that no common variants effect is identified for this pathway. For the pathways with selected common variants, we then define a risk score as the summation of risk alleles (risk alleles are determined through a univariate association analysis on each SNP). This risk score differs from the risk score for rare variants in that effect directions have been accounted for [9]. We denote this risk score as RS_common_.

**Step 3.** Apply both the unadjusted and adjusted logistic regression models to the combined rare and common variants effect. The combining is done by using a weighted summation of the risk scores for rare and common variant effects. To decide the weights, we dichotomize the rare and common variant risk scores using their medians and then fit logistic regression models on the dichotomized variables to estimate the odds ratios. We then use the estimated odds ratios as the weights. Specifically, the final risk score for a given pathway is:(4)

where OR*_r_* is estimated from:(5)

and equals exp; sign(OR*_r_* − 1) is 1 if OR*_r_* > 1 and 0 otherwise. *I*(·) is an identity function. The definitions of the common variant risk component are similar.

**Step 4.** Correct for inflated type I error. We realized that the pathway effect selected from step 3 may have inflated type I error as a result of model selection. The asymptotic *p*-value based on standard normal distribution of the *Z* statistics does not consider such inflated type I error. To evaluate the pathway effect, we generate an empirical distribution based on the *Z* statistics of testing RS_final_. Such empirical distribution use all the *Z* statistics from step 3, except the top *k* pathway *Z* statistics (i.e., *k* = 10). Then we estimate empirical *p*-values for the *Z* statistics of the top *k* pathway using this empirical distribution. Such empirical *p*-values can be used to evaluate whether the top pathway effects satisfy the same distribution as the rest of the pathway effect.

Pathways information is obtained in two steps: (1) by mapping all SNPs to genes based on the SNP information provided by GAW17; and (2) by mapping genes to pathways, where genes resulting from step 1 had been identified in predefined gene sets or pathways (http://www.broad.mit.edu/gsea/msigdb/index.jsp). To ensure that each pathway has a reasonable number of variants, in the analysis we use only gene sets or pathways with at least 5 genes (1,126 pathways).

We perform additional analyses to detect the difference of disease prevalence across different populations (Asian, African, and European) to find out whether the nature of the disease is population specific. For those pathways identified as associated with the disease status, we use a linear regression model to assess whether the genetic risk score across different populations is different. We apply a multivariate model to disease status on population and to those identified pathway risk scores that have a different profile across population. We apply the likelihood ratio test to test whether the population effect remains significant.

## Results

### Quality control

We removed 1,314 SNPs for which the HWE test *p*-value was smaller than 1 × 10^−6^. This left us with 23,173 autosomal SNPs and 697 samples for the follow-up analysis. Univariate modeling for the effect of a single SNP yielded no significant signal at an adjusted significance level of 0.05/24,487 = 2.0419 × 10^−6^ after all clinical factors (Age, Sex, Smoking status, Population) were accounted for.

### Pathway analysis

Of the 1,126 pathways, 772 had at least one common variant that was selected by the LASSO. Of these, 152 pathways had significant common variant effects (*p* < 6.47 × 10^−5^, corresponding to a Bonferroni-corrected significance level of 0.05). In comparison, only two pathways reached Bonferroni-adjusted significance (*p* = 1.74 × 10^−5^ and 4.78 × 10^−5^, respectively) for their rare variant effects. The strongest signal of rare variant effect came from a pathway known as PENG_GLUTAMINE_UP (*p* = 1.74 × 10^−5^, OR = 1.422); however, significant effects from common variant(s) of this pathway were only suggestive (*p* = 0.01, OR = 1.16).

When common and rare variant effects were combined, 162 out of the 772 pathways had a suggestive signal (unadjusted *p* < 6.47 × 10^−5^). There was a leap in *p*-values assessed through combined effects (unadjusted) between the seventh and eighth most significant pathways, which led us to assess empirical *p*-values for the top seven pathways. Statistics directly from the model for the seven pathways are given in Table 1. Rare variants generally had a much weaker signal than common variants within these pathways, and the combined effects were dominated by those from common variants. In addition, all seven top-ranked pathways showed stronger genetic effects after clinical factors were adjusted. It was interesting that these pathways all contained some true genes or SNPs (Table 2). This might have been partly driven by the size of the pathways (number of genes or SNPs they contained). However, our algorithm identified large pathways as well as some small pathways that contained more true genes or SNPs.

**Table 1:** *p*-Values and ORs for different effects

					Combined effects			
			
	Rare variant effects, unadjusted		Common variant effects, unadjusted		Unadjusted		Adjusted by clinical factors^a^	
	
Pathway	*p*-value	OR	*p*-value	OR	*p*-value	OR	*p*-value	OR
STEMCELL_EMBRYONIC_UP	0.001	1.09	2.28 × 10^−20^	1.34	1.65 × 10^−19^	1.06	4.83 × 10^−22^	1.1
LEE_TCELLS2_UP	0.012	1.06	7.44 × 10^−19^	1.33	2.74 × 10^−16^	1.05	2.09 × 10^−21^	1.09
DAC_PANC_UP	0.39	1.06	3.44 × 10^−16^	1.3	1.72 × 10^−15^	1.07	1.79 × 10^−17^	1.12
AGED_RHESUS_UP	0.003	1.24	1.02 × 10^−12^	1.33	2.51 × 10^−13^	1.09	1.79 × 10^−15^	1.15
ICHIBA_GVHD	0.287	1.09	3.56 × 10^−13^	1.41	1.18 × 10^−12^	1.12	8.53 × 10^−14^	1.16
BRCA_ER_NEG	0.009	1.07	5.40 × 10^−11^	1.22	4.48 × 10^−12^	1.08	9.94 × 10^−17^	1.13
HSC_HSC_ADULT	0.018	1.11	4.26 × 10^−12^	1.29	5.84 × 10^−12^	1.09	1.49 × 10^−12^	1.14

^a^ Clinical factors include age, sex, smoking status, and ethnic origin.

**Table 2:** Pathway size and number of true genes or SNPs that each pathway contains

Pathway	Number of genes	Number of true genes	Number of rare SNPs	Number of true rare SNPs	Number of common SNPs picked by LASSO	Number of true common SNPs
STEMCELL_EMBRYONIC_UP	181	1	1,046	3	53	1
LEE_TCELLS2_UP	169	3	1,114	4	51	1
DAC_PANC_UP	65	2	504	9	39	0
AGED_RHESUS_UP	50	2	392	10	24	3
ICHIBA_GVHD	43	3	352	5	18	2
BRCA_ER_NEG	152	2	791	4	33	1
HSC_HSC_ADULT	56	2	408	11	27	0

Based on the empirical *p*-values, only the top three pathways (*p* = 4.51 × 10^−5^, 3.76 × 10^−6^, 3.06 × 10^−6^) reached a Bonferroni-corrected significance level (*p* < 6.47 × 10^−5^).

### Population structure

**Figure 1:**
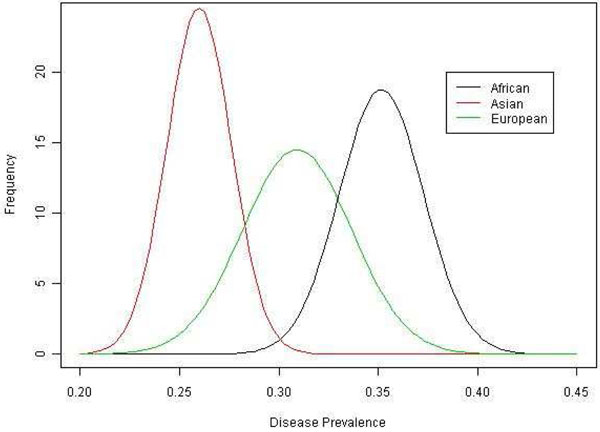
Disease prevalence distribution in different population groups (based on 200 replications)

The disease prevalence in different populations was significantly different (*p* < 2.2 × 10^−16^) (Figure 1). The African cohort had the highest risk, followed by the European cohort; the Asian cohort had the lowest risk. Linear regression models that explored associations of genetic risk (combined risk score) and population showed highly significant differences for the seven most significant and disease-associated pathways (all *p*-values less than 1 × 10^−10^). Likelihood ratio tests that assessed the effects of population on disease when genetic risk scores were taken into account gave *p*-values greater than 0.05 for five of the seven pathways, which suggests that prevalence differences in different populations are likely due to genetic differences.

## Discussion and conclusions

In this study, we evaluated the genetic effect on the pathway level based on both common and rare genetic variants. Overall, we did not find significant associations at the single-SNP level; however, several significant pathways (of either rare variants alone or rare and common variants together) were detected to be potentially associated with disease status. The associations still exist after adjusting for other factors, which is robust evidence for the pathway-based effect. Because of this result, we believe that modeling SNPs at the pathway level can enhance the power to detect genetic effects compared to modeling single SNPs. In common practice, investigators may have to take multiple steps to make good use of pathway information. These steps include using different methods to screen out potential pathways in the first stage and then validating those results.

The top identified pathways show that both common and rare variants have consistent signals but with different magnitudes. For this study, the common variants showed stronger signals. This suggests that the underlying mechanism for genetic effects is likely to be a joint effect consisting of both common and rare variants.

The significant difference of the disease prevalence across subpopulations is interesting and led us to explore potential factors affecting it. Using the likelihood ratio test with the genetic risk score in a nested model, we found that the disease risk was no longer significantly associated with population category. This suggests that the genetic component may partly explain this difference in disease prevalence. Different distributions of the disease-related genetic profiles may cause different disease prevalences across populations. For example, the African cohort has the highest genetic risk score, the European cohort has an intermediate risk score, and the Asian cohort has the lowest risk score, corresponding to the highest disease risk in the African cohort, an intermediate risk in the European cohort, and the lowest risk in the Asian cohort. Further analysis may be needed to identify other effects that may contribute to the different disease prevalences across the subpopulations.

## Competing interests

The authors declare that they have no competing interests.

## Authors’ contributions

LC carried out the analysis and drafted the manuscript. PZH prepared the pathway data. WX designed the analysis plan. All authors participated in discussions of analysis plan and helped revise the manuscript.
